# Functional Analysis of Organic Acids on Different Oilseed Rape Species in Phytoremediation of Cadmium Pollution

**DOI:** 10.3390/plants9070884

**Published:** 2020-07-13

**Authors:** Shi Li, Sixiu Le, Xin Wang, Jiuyuan Bai, Rui Wang, Yun Zhao

**Affiliations:** Key Laboratory of Bio-Resource and Eco-Environment of Ministry of Education, College of Life Sciences, Sichuan University, No. 24 South Section 1, Yihuan Road, Chengdu 610065, China; 2017222040105@stu.scu.edu.cn (S.L.); 2017322040057@stu.scu.edu.cn (S.L.); 2018322040056@stu.scu.edu.cn (X.W.); 2018322040035@stu.scu.edu.cn (J.B.); zhaoyun@scu.edu.cn (Y.Z.)

**Keywords:** antioxidant enzyme, cadmium, oilseed rape, organic acids, phytoremediation, combined remediation, soil pollution

## Abstract

Cadmium (Cd) pollution in soil is becoming increasingly serious due to anthropogenic activities, which not only poses a threat to the ecological environment, but also causes serious damage to human health via the biological chain. Consequently, special concerns should be paid to develop and combine multiple remediation strategies. In this study, different subspecies of oilseed rape, *Brassica campestris,* *Brassica napus* and *Brassica juncea* were applied, combined with three organic acids, acetic acid, oxalic acid and citric acid, in a simulated Cd-contaminated soil. Various physiological and biochemical indexes were monitored in both plant seedling, growth period and mature stage. The results showed that organic acids significantly promoted the growth of *Brassica campestris* and *Brassica juncea* under Cd stress. The photosynthesis and antioxidant enzyme activities in *Brassica campestris* and *Brassica juncea* were induced at seedling stage, while that in *Brassica napus* were suppressed and disturbed. The enrichment of Cd in oilseed rape was also obviously increased. *Brassica juncea* contained relatively high resistance and Cd content in plant but little Cd in seed. Among the three acids, oxalic acids exhibited the most efficient promoting effect on the accumulation of Cd by oilseed rape. Here, a comprehensive study on the combined effects of oilseed rape and organic acids on Cd contaminated soil showed that *Brassica juncea* and oxalic acid possessed the best effect on phytoremediation of Cd contaminated soil. Our study provides an optimal way of co-utilizing oilseed rape and organic acid in phytoremediation of Cd contaminated soil.

## 1. Introduction

Soil pollution is becoming increasingly fierce with the development of industry, agriculture and urbanization on a global scale. Inorganic contaminants (mainly heavy metals, HMs) concentrated in soil has aroused major concerns in line with a growing number of reports about heavy metal pollution emerged in an endless stream. For example, 82.8% soil contamination is caused by inorganic contaminants in China while 34.8% present in soil and groundwater in Europe [1,2]. Compared with diverse soil heavy metal pollution elements, cadmium (Cd) is the most serious one and contributes 7% to the HMs contaminated soil in China [3]. Cd is generally considered as a kind of highly mobile, toxic, easily absorbed elements by plants and is difficult to degrade [4,5]. It is commonly understood that Cd is not an essential element and does not have any physiological function in plants. Even excessive Cd in plants destroys not only the structure of plant cells, but it also disturbs the normal physiological process, especially photosynthesis and antioxidant system, ultimately affecting plant growth and production [6]. Increasing evidence shows that Cd is highly pathogenic and carcinogenic when accumulated in the human body through the food chain [7].

**Figure 1:**
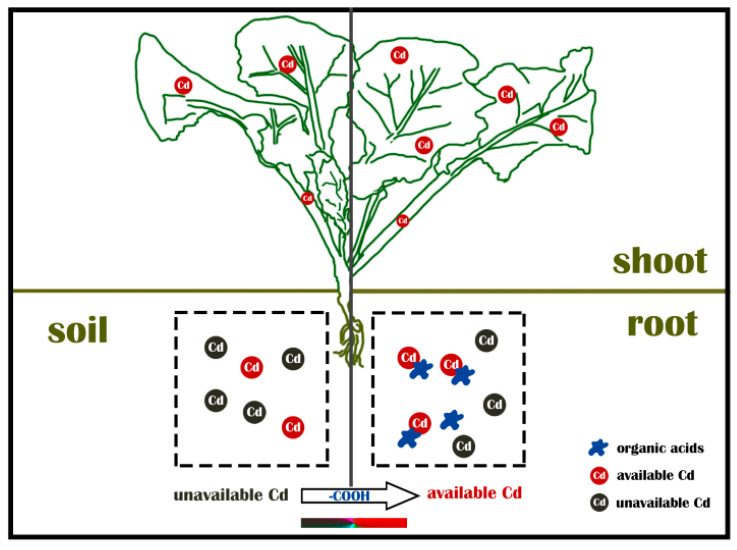
Pattern diagram of organic acids promoting Cd absorption in oilseed rape. In comparison to Cd contaminated soil without application of organic acids (**left**), the addition of organic acids can promote the conversion of unavailable Cd to available Cd so that they enhance the absorption in oilseed rape.

Physical repair, chemical control and bioremediation are current methods for soil heavy metal pollution remediation [8]. Due to the inexpensive and high-efficiency features on heavy metal remediation, chemical method is prevalently utilized in practice. However, it has certain limitations and easily causes secondary pollution to the soil. Hence the phytoremediation, a kind of cost-effective and environment friendly method, is of great concern. However, due to the complexity of soil heavy metal contaminants and the diversity of the environment, single repair method is incapable of achieving the expected result [9].Thus the joining of multiple methods has become the mainstream of soil remediation. Organic acids secreted by plant root (especially in hyperaccumulators) can form metal ligand complexes under HMs stress and participate in the process of heavy metal absorption, transportation and accumulation [10]. It has been proven that the fraction and bio-availability of Cd are closely correlated with the pH value in soil [11]. Reduced pH value makes heavy metal easily turn into a soluble complex and increases its bioavailability, which consequently affects the absorption of heavy metals by plant [10,12]. Therefore, the application of organic acids to improve the phytoremediation could be a quick and efficient approach. The facilitating effect of exogenous organic acids on Cd accumulation by oilseed rape is depicted in the schema graph (Figure 1). Brooks et al. [13] first proposed the concept of hyperaccumulator. So far, hyperaccumulation plants have been reported more than 400 kinds, which were widely distributed in more than 50 families, especially in Brassicaceae [12]. Kumar et al. [14] revealed that *Brassica juncea* is a Cd super-enriched plant. Likewise, Fei et al. [15] showed that *Brassica juncea* had high enrichment for heavy metal Cd. Since then, special attentions have been paid to *Brassica* [16]. Oilseed rape, which belongs to *Brassica*, is one of the most widespread oil crops in China. It has a long history of cultivation and abundant genetic diversity. Oilseed rape is commonly divided into three types: *Brassica campestris*, *Brassica napus* and *Brassica juncea*. Recent studies have revealed that not only *Brassica juncea*, but also *Brassica napus* and *Brassica campestris* processes the ability to enrich Cd. However, fewer investigators pay close attention to the comparison among the three types of oilseed rape let alone with the application of organic acids.

In this study, we investigated the growth status, biochemical response and Cd accumulation of three oilseed rape subspecies (*Brassica campestris*, *Brassica napus* and *Brassica juncea*.) with the treatment of three different organic acids (oxalic acid, acetic acid and citric acid).

## 2. Materials and Methods

### 2.1. Soil, Plant and Organic Acids 

The soil used in this experiment was purchased from Sansheng Town, Chengdu City, Sichuan Province. The soil was air-dried under natural conditions and used for pot experiment after stones and other debris were filtered out. The basic physicochemical properties and Cd concentration were determined and shown in Table 1. Three varieties of oilseed rape, *Brassica campestris* (15–1593), *Brassica napus* (15–1453) and *Brassica juncea* (Deyangjie) were planted in the experiment, which were held by our laboratory. Oxalic acid, acetic acid and citric acid were selected as exogenous additives in present study.

### 2.2. Experiment Design 

Prior to the cultivation of oilseed rape, Cd applied as CdCl_2_ was added to the soil up to the final Cd concentration to 20 mg/kg at three intervals and stabilized at room temperature for 30 days. The pot used in our study was 21 cm in diameter and 10 cm in height, which can hold 1 kg soil per pot. Seeds of every variety were selected with no insect pests, in full and uniform grain size. After surface sterilization and water immersion for 24 h, seeds were sown in the pots uniformly. When seeds germinated and grew one true leaf, the seedlings were thinned to leave five plants with the same growth status per pot. Thereafter, the solution of oxalic acid, acetic acid and citric acid were added to corresponding treatment group to the final concentration 3 mmol/kg, 4 mmol/kg and 2 mmol/kg [17] in the soil respectively with free treatment group as control. The same amount of organic acid solution was replenished once a month. Each treatment was performed in triplicate and regularly irrigated by dripping to ensure plants water demand. The whole planting period lasted from November 15th in 2017 to April 15th in 2018 in outdoors. 

### 2.3. Sample Collection

Growth period samples were collected carefully at 75 days after germination of oilseed rape and rinsed with deionized water thoroughly. Then plant materials were divided into two parts (roots and shoots). The roots of all samples were immersed in 200 mM Na_2_-EDTA 15 min, then washed with tap and deionized water for five times in order to remove any extracellular Cd adsorbed on the root surface. Part fresh leaves for further biochemical characterization were put into −80 °C refrigerator after freezing in liquid nitrogen immediately. Other samples were first stored at 105 °C for 10 min, and then dried in a 60 °C oven until constant weight. Mature samples were harvested on April 15th in 2018. Then the entire plant was divided into root, shoot, silique sheath and seed. In the same way as above, all plant samples were dried to constant weight. In the above process, the DW (dry weight) and plant height of all samples along with root length in seedlings were recorded synchronously.

### 2.4. Soil Physicochemical Analysis

In order to indicate the basic physicochemical property of the soil used in present study, we measured several general indicators containing pH, total nitrogen (TN), total phosphorous (TP), total potassium (TK), soil organic matter (SOM) and cadmium concentration. pH value was measured in a 1:2.5 (*w*/*v*) soil/decarbonized water suspension liquid via an PHSJ-3F pH meter (Shanghai INESA Instrument, Shanghai, China). The content of organic matter and TN was both followed the method described by Zhang et al. [18]. The TP content was detected through the ammonium molybdate spectrometry method described by Jacobson and Lockitch [19]. The measurement of TK was referred to the method introduced in Reference [20]. 

The determination of Cd content in plant and soil samples was referred to national standard (GB/T 17141-1997) in China with slight modification. As for plant samples, 0.2-g abovementioned dry plant powders were put into 100-ml polytetrafluoroethylene crucibles and digested with the mixture of high purity concentrated HNO3 and HCLO4 (5:1) in a graphite digestion apparatus, where-after, the mixture solution was diluted into a 50-mL volumetric flask with ultrapure deionized water, then stored in a refrigerator at 4 °C for further determination. Cd in the digestion solution was determined by inductively coupled plasma mass spectrometry (Thermo Fisher Scientific, Massachusetts, MA, USA). The national standard reference substance GBW07408 GSS-8 (standard substance for soil composition analysis) and GBW07603 (standard substance for composition analysis of shrub branches and leaves) was used for quality control during digestion and measurement. 

### 2.5. Translocation Factor

Translocation factor (S/R) is an indicator of the ability of plants to transport certain heavy metals from roots to aboveground part. S/R = the ratio of the metal content in the aerial part of the plants to the metal content in the underground part. 

### 2.6. Determination of Biochemical Parameters

Heavy metals are known to affect plant photosynthesis and antioxidant enzyme systems. The content of chlorophyll and activity of several antioxidant enzymes were detected in present study to reflect the effect of Cd and organic acids on the biochemical characteristics in oilseed rape. The quantification of total chlorophyll was conducted through acetone alcohol mixture method improved by Taghadomi et al. [21] for enzyme activity determination 0.1 g leaves of seedling was added into 1 ml pH 7.4 PBS extracting solution to obtain crude enzymes. The activities of catalase (CAT), peroxidase (POD), superoxide dismutase (SOD) and the content of malondialdehyde (MDA) in the acquisition were determined by corresponding enzyme activity kit (spectrophotometry) bought from COMIN Biotechnology Co. Ltd. in Suzhou, China.

### 2.7. Statistical Analysis

All data shown in the figures were the mean of three replicated independent determination and statistically analyzed using IBM SPSS Statistics 19.0 (IBM Inc., New York, NY, USA), and Two-way ANOVA was used to analyze the data significance in the article [22]. The figures were drawn by Graphpad Prism 7 software (Graphpad Software Inc., San Diego, CA, USA).

## 3. Results

### 3.1. Effect of Cadmium Combined with Organic Acids on the Growth of Rapeseed

**Figure 2:**
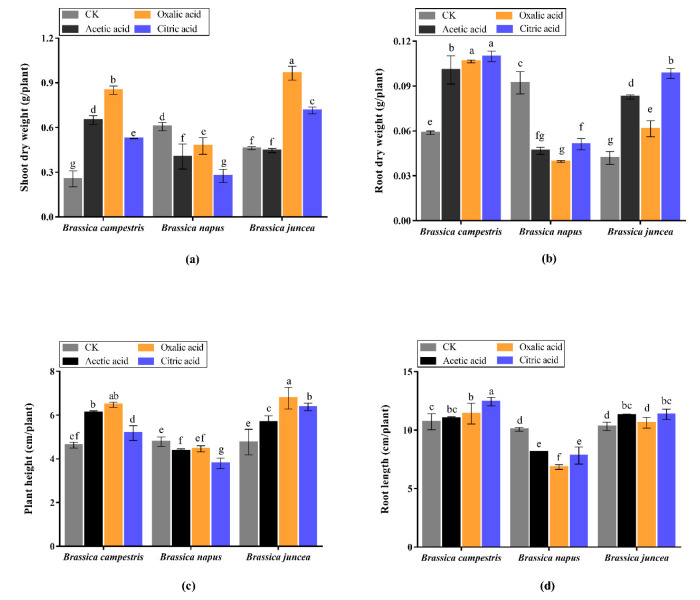
Seedling growth for the three *Brassica* species in presence of 20 mg/kg Cd and following organic acid treatment. The Values are means ± SE from three biological replicates. Values followed by different letters indicate statistical significance based on two-way ANOVA (*p* < 0.05). (**a**) Shoot dry weight. (**b**) Root dry weight. (**c**) Plant height. (**d**) Root length.

The growth of three varieties of oilseed rape in seedling stage is shown in Figure 2. In the absence of organic acid treatment (Control), the plant DW in *Brassica campestris* and *Brassica juncea* was apparently higher than *Brassica napus*, which indicated a stronger growth capacity and higher biomass in the two varieties. However, differing from the significant inhibiting effect on *Brassica napus*, the exogenous application three organic acids promoted plant growth of *Brassica campestris* and *Brassica juncea* to different degree, especially in total plant height. Compared with *Brassica campestris* and *Brassica juncea*, three organic acids all could greatly increase plant dry weight in *Brassica campestris*, while only oxalic acid held the capacity to significantly enhance both two indexes in *Brassica juncea*, in which the root dry weight and plant height were notably increased by each organic acid. In addition, there were some commonalities exhibited in the diverse varieties. It can be seen that among all organic acids, oxalic acid showed the most obvious promoting effect except for *Brassica napus*. Moreover, the root length in all groups was relatively stable compared to other indexes, with or without addition of organic acids.

**Figure 3:**
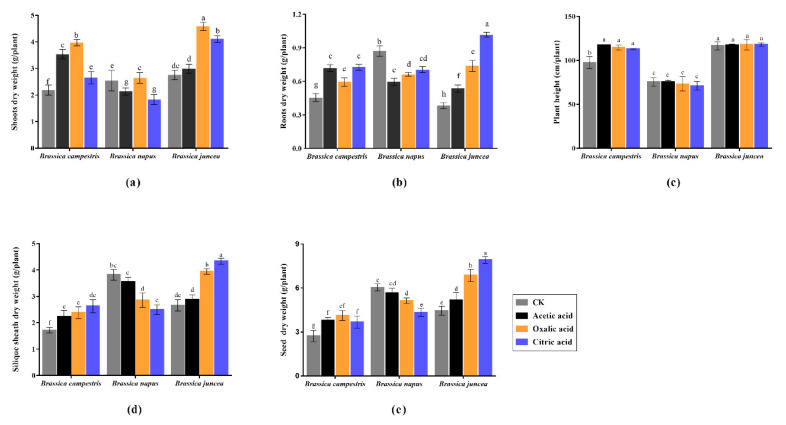
Plant characteristics at maturity stage of the three *Brassica* species grown in the presence of 20 mg/kg Cd and treated with organic acid. The Values are means ± SE from three biological replicates. Values followed by different letters indicate statistical significance based on two-way ANOVA (*p* < 0.05). (**a**) Shoot dry weight. (**b**) Root dry weight. (**c**) Plant height. (**d**) Silique sheath weight. (**e**) Seed weight.

The growth status of oilseed rape in maturity is depicted in Figure 3, including plant height, dry weight of roots, shoots, silique sheaths and seeds. The results show that all organic acids had no obvious effect on the plant height of *Brassica napus* and *Brassica juncea*, but significant enhancement occurred in *Brassica campestris* without overt difference among the three organic acids treatments. The addition of organic acid increased both shoot and root dry weight of *Brassica campestris* and *Brassica juncea*. There was no difference on shoots dry weight between oxalic acid treatment group and CK for *Brassica napus*, while distinct reduction was found in both acetic acid and citric acid group. Similar to the results in seedlings, inhibiting effect is also appeared in *Brassica napus* by organic acids under Cd stress. Seeds weight is a critical indicator for agricultural production. Consistent with plant dry weight (Figure 3a,b), exogenous addition of three kinds of organic acids (especially citric acid) dramatically increased silique sheaths weight and raised seeds production in *Brassica campestris* and *Brassica juncea*, the results of which are shown in Figure 3d,e. Inversely, significant reduction was observed when treated with oxalic acid and citric acid in *Brassica napus*. Overall, the results reveal that diverse responses occurred in various oilseed rape varieties when treated with different organic acids. The growth of *Brassica campestris* and *Brassica juncea* was promoted but inhibited on *Brassica napus*.

### 3.2. Effects of Cadmium Combined with Organic Acids on Plant Antioxidant System and Chlorophyll Content

**Figure 4:**
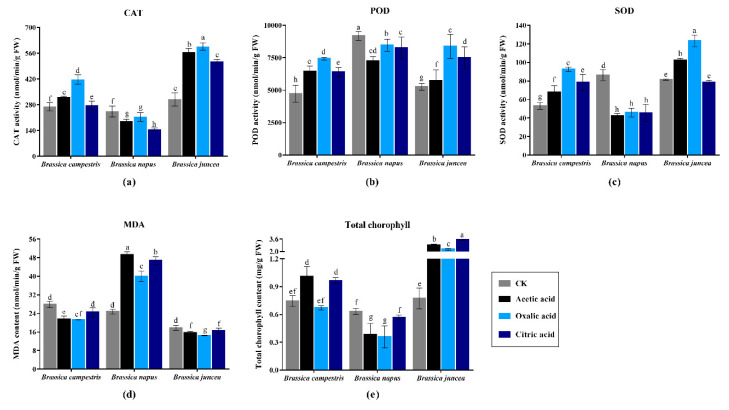
The antioxidant system in three *Brassica* species under 20 mg/kg Cd stress. The Values are means ± SE from three biological replicates. Values followed by different letters indicate statistical significance based on two-way ANOVA (*p* < 0.05). (**a**) Catalase CAT activity. (**b**) Peroxidase POD activity. (**c**) Superoxide dismutase SOD activity. (**d**) Malondialdehyde MDA content. (**e**) Total chlorophyll content.

The activities of CAT, SOD and POD in *Brassica campestris* and *Brassica juncea* were significantly higher than those in CK (Figure 4a–c), while these enzymes in *Brassica napus* were lower than CK. MDA content in leaves is an important indicator of the degree of membrane peroxidation in cells, which is positively correlated with the degree of environmental stress of plants. The content of MDA (Figure 4d) was obviously decreased in *Brassica campestris* and *Brassica juncea* as compared to control while the highest content of MDA was found in *Brassica napus*. The activities of CAT and SOD in *Brassica juncea* were significantly higher and the content of MDA was obviously lower than others. With regard to three organic acids, oxalic acid can effectively improve the activities of antioxidant enzymes and reduce the MDA content in *Brassica campestris* and *Brassica juncea*, which suggests that oxalic acid is more conducive to induce plant antioxidant system and effectively eliminate reactive oxygen species (ROS). We speculated that *Brassica juncea* has better adaptability to adverse environments than *Brassica campestris* and *Brassica napus*.

Chlorophyll is the critical material basis for photosynthesis, and its content is the main indicator for judging the photosynthesis intensity of plants [23]. It can be seen from the Figure 4e that the content of total chlorophyll in *Brassica campestris* is significantly higher as compared to CK when treated with acetic acid. For *Brassica napus*, there are no significant differences in citric acid treatment compared with CK, whereas distinctly reduction is emerged when treated with acetic acid and oxalic acid. Surprisingly, the chlorophyll content in *Brassica juncea* exhibited extremely significant enhancement which is clearly distinct from *Brassica campestris* and *Brassica napus* and citric acid performed the most obvious inductive effect. We herein attribute this to the higher photosynthetic efficiency in *Brassica juncea* which may be extended to *Brassica juncea.*

### 3.3. Effects of Organic Acids on the Cadmium Accumulation

**Figure 5:**
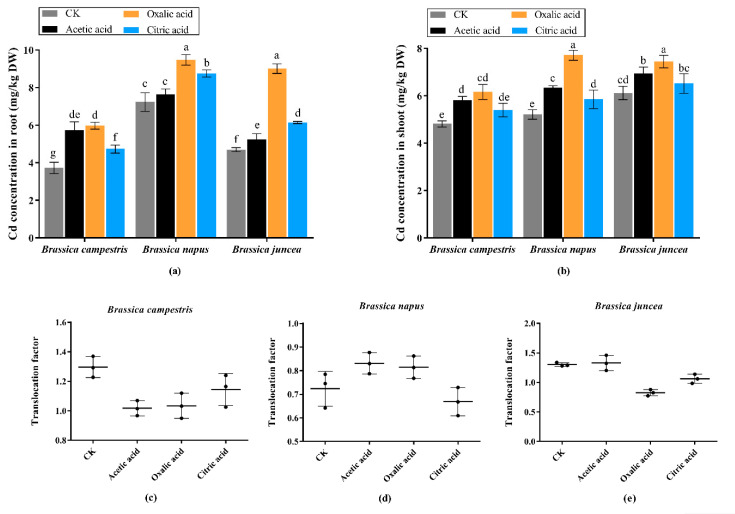
Cd concentration in three *Brassica* species at seedling stage. The Values are means + SE from three biological replicates. Values followed by different letters indicate statistical significance based on two-way ANOVA (*p* < 0.05). (**a**) Cd concentration in root. (**b**) Cd concentration in shoot. (**c**) Cadmium translocation factor in *Brassica campestris*. (**d**) Cadmium translocation factor in *Brassica napus*. (**e**) Cadmium translocation factor in *Brassica juncea*.

The growth period samples were divided into root and shoot respectively. Dried samples were ground thoroughly and sieved by a 60-mesh sifter prior to the determination of Cd concentration. As shown in Figure 5, it can be seen that *Brassica napus* can absorb more Cd in root while it is more abundant in the shoot of *Brassica juncea* without application of organic acids. However, significant increase was observed in all treatments with the addition of organic acids. The highest concentration of Cd appears in the root of the *Brassica napus* and *Brassica juncea*, reaching 9.264 mg/kg and 9.011 mg/kg, respectively. The concentration of Cd in the leaves also reached up to 7.710 mg/kg and 7.441 mg/kg in *Brassica napus* and *Brassica juncea*. Overall, *Brassica napus* and *Brassica juncea* have stronger enrichment ability for Cd in contrast to *Brassica campestris*. Meanwhile, three kinds of organic acids all have the ability to promote the accumulation of Cd in plant root and shoot, and oxalic acid in particular shows better efficiency. The Cd translocation factor in oilseed rape materials is described in Figure 5c–e. Three kinds of organic acids all can reduce the translocation factor of Cd in *Brassica campestris*. A similar case was also observed in *Brassica juncea*, but only oxalic acid and citric acid significantly reduced the translocation factor. Contrary to the conditions in *Brassica campestris* and *Brassica juncea*, acetic acid and oxalic acid significantly increased Cd translocation factor in *Brassica napus*. 

**Figure 6:**
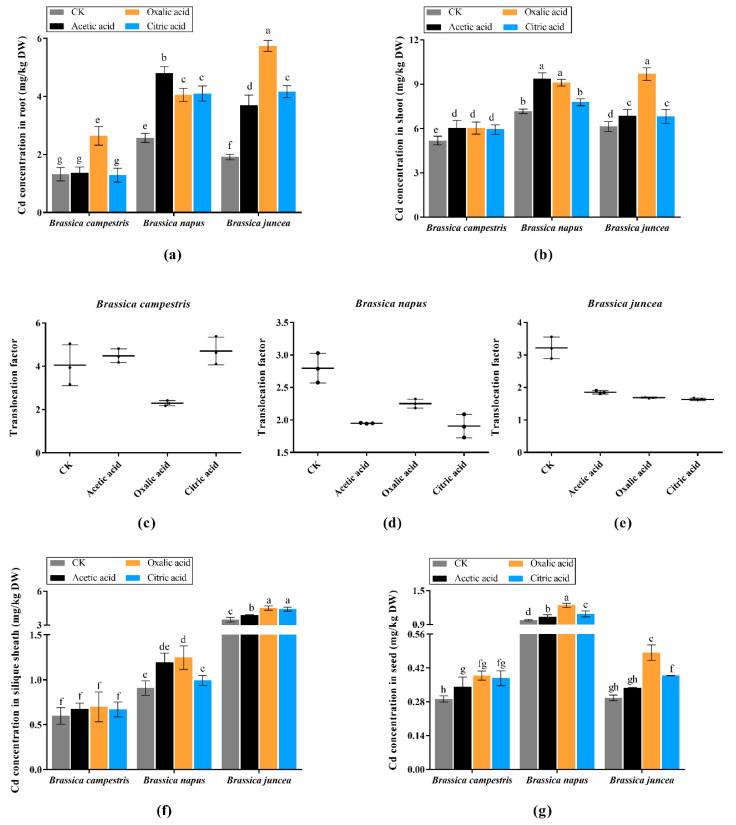
Cd concentration in three Brassica species at maturity stage. The Values are means ± SE from three biological replicates. Values followed by different letters indicate statistical significance based on two-way ANOVA (*p* < 0.05). (**a**) Cd concentration in root. (**b**) Cd concentration in shoot. (**c**) Cadmium translocation factor in *Brassica campestris*. (**d**) Cadmium translocation factor in *Brassica napus*. (**e**) Cadmium translocation factor in *Brassica juncea*. (**f)** Cd concentration in silique sheath. (**g**) Cd concentration in seed.

Cd content in the root, shoot, silique sheath and seed of mature oilseed rape materials is shown in Figure 6. Similar to the result in seedlings, *Brassica napus* and *Brassica juncea* possesses higher ability to enrich Cd in root and shoot. In addition, the effect of organic acids on *Brassica napus* and *Brassica juncea* is also more obvious. Three organic acids can promote the absorption of Cd in root and acetic acid has the most obvious effect on *Brassica napus* up to 4.801 mg/kg and oxalic acid on *Brassica juncea* reaching to 5.736 mg/kg. In *Brassica campestris*, only oxalic acid escalates the Cd concentration in root reaching to 2.639 mg/kg. In shoot (Figure 6b), organic acids have promoting effect on *Brassica campestris* without obvious divergence among these acids. For *Brassica napus*, both acetic acid and oxalic acid perform excellent promoting effect and achieved to 9.375 mg/kg and 9.107 mg/kg respectively. Oxalic acid has increased significantly the absorption of Cd in the shoot with absorption of 9.687 mg/kg for *Brassica juncea*. As shown in Figure 6c–e, Except *Brassica campestris*, *Brassica napus* and *Brassica juncea* Cd translocation factors are reduced after addition with all of three organic acids. The concentration of Cd in silique sheath is shown in Figure 6f, where there is no huge difference in *Brassica campestris* and *Brassica napus* compared with CK, while significant increase can be found in *Brassica juncea* with every organic acid treatment. Figure 6g shows the Cd concentration in seed. Interestingly, through high concentration of Cd presents in *Brassica juncea*, the content of Cd in seed shows quite a low level which is in contrast to *Brassica napus*. *Brassica campestris* accumulates the relatively minimal Cd in silique sheath and seed among three varieties. The oxalic acid is the most effective additive leading to higher Cd in seed compared with the other two organic acids. The most abundant Cd concentrations all occurs in the oxalic acid treatment, reaching 0.389 mg/kg in *Brassica campestris*, 1.238 mg/kg in *Brassica napus* and 0.484 mg/kg in *Brassica juncea*. Based on the Cd safety threshold (0.5 mg/kg) of similar crop peanut prescribed in GB2762-2005 in China, the Cd content in the seed of *Brassica campestris* and *Brassica juncea* is within the limit, which suggests a relatively safe value. In our study, we concluded that *Brassica juncea* can absorb more Cd under oxalic acid with fewer Cd in seed.

## 4. Discussion

### 4.1. Effect of Organic Acids and Cadmium on the Growth of Rapeseed

A lot of research efforts have shown that high concentration of Cd is deleterious to plants, such as destruction of plant cell structure [24], inhibition of plant photosynthesis [25] and disturbance on plant nutrient absorption [26], etc. The limitation of Cd on the growth of plants through a series of physiological and biochemical activities and finally exhibits on the plant growth status such as height and biomass. In heavy metal contaminated soil, diverse plants species and varieties have different tolerance, and present divergent responses to Cd stress. Mohamed et al. [27] revealed that fresh weight, dry weight and root length of *Brassica juncea* were reduced under Cd stress. Liu et al. [28] indicated that organic acids can activate heavy metals in the soil and aggravate the stress of Cd on plants. Generally speaking, a plant with high tolerant capability to heavy metals can normally grow in contaminated soil and the tolerance of plants can be evaluated by their morphological parameters [29,30]. In this study, three organic acids could significantly enhance biomass of *Brassica campestris* and *Brassica juncea*, which indicated that *Brassica campestris* and *Brassica juncea* had higher resistance and can be induced by organic acids under a certain concentration of stress. In contrast, the diminishment of biomass in *Brassica napus* by adding organic acid indicated that with the higher concentration of Cd (20 mg/kg), and the toxic action poisoned by the activated heavy metal ions were beyond its bearing capacity in *Brassica napus*. Among all three organic acids, oxalic acid showed the strongest effect. This phenomenon may be attributed to the activation of nutrient elements thus promoting the absorption of nutrients by plants within the resistance of Cd in respective plant. In terms of seed yield, organic acids were more effective in *Brassica juncea* in contrast to *Brassica campestris*. It can be deduced that *Brassica juncea* can better adapt to acidic soil conditions, while an acidic environment possibly militated against the fructification of *Brassica campestris*. This pattern was also supported by the result of chlorophyll content (Figure 4). It was stated that chlorophyll is the most basic substance in photosynthesis for plants and directly reflects the intensity of photosynthesis. Cd affects plant photosynthesis mainly by affecting the chlorophyll content in plants [31]. Chlorophyll content in *Brassica juncea* was significantly higher than that in other two oilseed rape under organic acids treatment, which was attributed to its high seed yield.

### 4.2. Effects of Organic Acids and Cadmium on Antioxidant System in Rapeseed

Unfavorable environment can cause free radicals directly or indirectly during plant growth and metabolism, which can cause toxic effects on plant cell membranes [32]. The antioxidant enzyme system can effectively remove active oxygen free radicals in plants and protect plants. In the same way, Cd stress can cause severe oxidative stress [33] and produce numerous free radicals in plants [34], which can destroy plant cell membranes. Antioxidant enzyme system can effectively remove active oxygen free radicals in plants and validly protect plants. Santos et al. [35] found that Cd stress increased the activity of CAT, SOD and APX enzymes in pepper leaves. Agrawal and Mishra [36] also found that the presence of Cd increased the activity of SOD and POD in pea seedlings but decreased the activity of CAT. In our results, *Brassica napus* showed high SOD and POD enzyme activity under Cd stress in the absence of organic acids, but MDA content went up dramatically once treated with organic acids. It can be explained by the statement from that organic acids interact with heavy metal ions in soil, and then can activate and increase their bioavailability [37]. In contrast to the increase of MDA, the enzyme activity of CAT, SOD and POD all significantly decreased. It can be deduced that the tolerance to Cd stress in *Brassica napus* was relatively defective. Especially after the application of organic acids, activated Cd ions brought more serious oxidative stress so that produced more deleterious damage on antioxidant system in *Brassica napus*. At the same condition, the MDA content still remained a relatively low level even if treated with organic acids in *Brassica campestris* and *Brassica juncea*. Moreover, the addition of organic acids in turn enhanced their enzyme activities, which strongly proved their high resistance on Cd stress and possessed adequate ability to eliminate oxygen free radicals so as to protect plant from the infringement. By measuring the antioxidant enzyme system function of three rapeseed varieties Cd stress, the results showed that *Brassica juncea* had the strongest tolerance to Cd.

### 4.3. Effect of Organic Acids on the Absorption of Cadmium by Rapeseed 

Heavy metal pollution in the soil is a widespread phenomenon, especially Cd pollution, which seriously affect the development of agriculture, so it is urgent to seek effective repairing methods [38]. Phytoremediation is now the mainstream of soil remediation, and the single repairing method has its limitations [39]. Therefore, this study uses a chemical-plant joint repairing method to promote the repairing effect. Former studies have shown that organic acids can activate heavy metals in the soil and allow them to be easily absorbed by plants [40]. In this study, by measuring the content of cadmium in rapeseed treated with organic acid, we analyzed the effect of organic acids on the absorption of Cd by rapeseed. compared with the control, the absorption of Cd increased in all tissues of the plant whether in seedling stage, growth period or mature period. It may be attributed to the activation of Cd by organic acids and consequently increase the bioavailability. In the growth period, *Brassica napus* performed higher Cd enrichment capability, especially when treated with oxalic acid. Differ from *Brassica napus*, the ability on the accumulation of Cd was relatively insufficient in growth period as it was in mature plant of *Brassica campestris*. The addition of organic acids significantly increased the content of Cd in both root and shoot of *Brassica napus* and *Brassica juncea*. In addition, compared with other two varieties, Cd enrichment in silique sheath was much higher in *Brassica juncea*, especially when treated with oxalic and citric acids. However, the Cd content in seed was extremely low, which may be a self-protective mechanism for plants to avoid the effects of Cd on their offspring. Quite the reverse, this feature did not occurre in *Brassica napus*. Large amounts of Cd were transported from the silique sheaths to the seeds leading to serious food safety risks in *Brassica napus*, which exceeded the Cd safety threshold of 0.5 mg/kg as specified by the similar crop peanut. The concentration of Cd in *Brassica campestris* was at a lower level in various treatments, which indicated that this kind of *Brassica campestris* is a low-accumulation variety that is endowed with good resistance of Cd. The most exciting implications were that although *Brassica juncea* enriched a high level of Cd in plant, minimal Cd was transported to seeds, which could be an ideal plant material both for phytoremediation and popularizing cultivation. Compared with the other two acids, oxalic acid can activate cadmium in soil more efficiently, thus effectively promoting the accumulation of cadmium in oilseed rape, which showed huge potential as exogenous additive to restore contaminated soil combined with phytoremediation.

## 5. Conclusions

**Figure 7:**
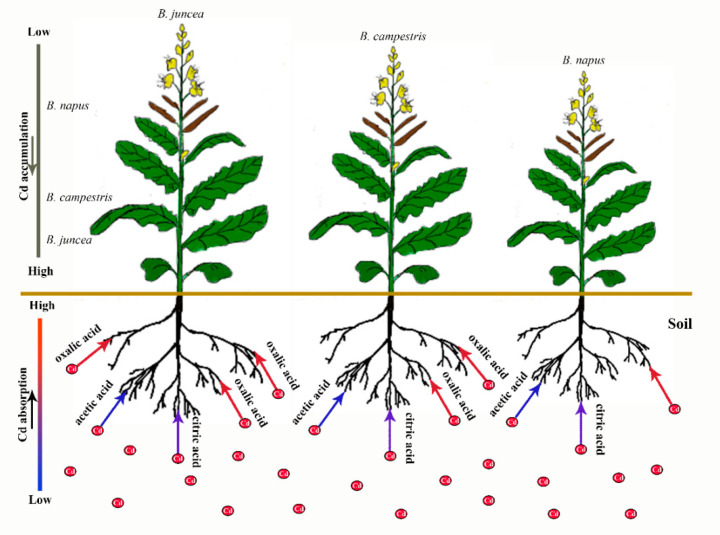
Pattern diagram of Cd phytoremediation by -utilizing *Brassica juncea* and oxalic acid. Arrows of different colors indicate the strength of Cd absorption mediated by organic acids.

In this study, we characterized the effects of three organic acids on the growth, antioxidant enzyme system and Cd accumulation in three varieties of oilseed rape. Overall, *Brassica juncea* and *Brassica campestris* possessed stronger resistance to Cd stress. Their growth was improved when treated with organic acids along with the induction of their antioxidant enzyme activities. But inhibition effect was performed in *Brassica napus*, and most Cd was concentrated in the shoot and seed along with the effect of organic acids. *Brassica juncea* could be an ideal plant material due to its high resistance and accumulation of Cd in plant (except seed) with the treatment of organic acids. Among the three acids, oxalic acids exhibited the most efficient promoting effect on the accumulation of Cd by oilseed rape. So, it can be a practicable combination for phytoremediation by use of *Brassica juncea* and oxalic acid (Figure 7).

## Figures and Tables

**Table 1:** Basic physicochemical properties of soil.

Ph	Organic Matter (G/Kg)	Total Nitrogen (G/Kg)	Total Phosphorous (G/Kg)	Total Potassium (G/Kg)	Cd (Mg/Kg)
6.9 ± 0.20	308.59 ± 17.35	11.20 ± 0.26	2.46 ± 0.31	13.11 ± 0.56	3.45 ± 0.17

Values represent mean ± standard deviation (n = 3).
